# Frequent traces of EBV infection in Hodgkin and non-Hodgkin lymphomas classified as EBV-negative by routine methods: expanding the landscape of EBV-related lymphomas

**DOI:** 10.1038/s41379-020-0575-3

**Published:** 2020-06-01

**Authors:** Lucia Mundo, Leonardo Del Porro, Massimo Granai, Maria Chiara Siciliano, Virginia Mancini, Raffaella Santi, Lynnette Marcar, Katerina Vrzalikova, Federica Vergoni, Gioia Di Stefano, Gianluca Schiavoni, Giovanna Segreto, Noel Onyango, Joshua Akelo Nyagol, Teresa Amato, Cristiana Bellan, Ioannis Anagnostopoulos, Brunangelo Falini, Lorenzo Leoncini, Enrico Tiacci, Stefano Lazzi

**Affiliations:** 1 Section of Pathology, Department of Medical Biotechnology, University of Siena, Siena, Italy; 2 Section of Pathology, University of Florence, Florence, Italy; 3 BioMaterials Cluster, Bernal Institute, University of Limerick, Limerick, Ireland; 4 Ireland, Health Research Institute (HRI), University of Limerick, Limerick, Ireland; 5 Institute of immunology and Immunotherapy, University of Birmingham, Birmingham, UK; 6 Section of Haematology and Clinical Immunology, Department of Medicine, University and Hospital of Perugia, Perugia, Italy; 7 Department of Clinical Medicine and Therapeutics, Unit of Medical Oncology, University of Nairobi, Nairobi, Kenya; 8 Department of Anatomical Pathology, University of Charité Berlin, Berlin, Germany

**Keywords:** Molecular biology, Tumour virus infections

## Abstract

The Epstein–Barr virus (EBV) is linked to various B-cell lymphomas, including Burkitt lymphoma (BL), classical Hodgkin lymphoma (cHL) and diffuse large B-cell lymphoma (DLBCL) at frequencies ranging, by routine techniques, from 5 to 10% of cases in DLBCL to >95% in endemic BL. Using higher-sensitivity methods, we recently detected EBV traces in a few EBV-negative BL cases, possibly suggesting a “*hit-and-run*” mechanism. Here, we used routine and higher-sensitivity methods (qPCR and ddPCR for conserved EBV genomic regions and miRNAs on microdissected tumor cells; EBNA1 mRNA In situ detection by RNAscope) to assess EBV infection in a larger lymphoma cohort [19 BL, 34 DLBCL, 44 cHL, 50 follicular lymphomas (FL), 10 T-lymphoblastic lymphomas (T-LL), 20 hairy cell leukemias (HCL), 10 mantle cell lymphomas (MCL)], as well as in several lymphoma cell lines (9 cHL and 6 BL). qPCR, ddPCR, and RNAscope consistently documented the presence of multiple EBV nucleic acids in rare tumor cells of several cases EBV-negative by conventional methods that all belonged to lymphoma entities clearly related to EBV (BL, 6/9 cases; cHL, 16/32 cases; DLBCL, 11/30 cases), in contrast to fewer cases (3/47 cases) of FL (where the role of EBV is more elusive) and no cases (0/40) of control lymphomas unrelated to EBV (HCL, T-LL, MCL). Similarly, we revealed traces of EBV infection in 4/5 BL and 6/7 HL cell lines otherwise conventionally classified as EBV negative. Interestingly, additional EBV-positive cases (1 DLBCL, 2 cHL) relapsed as EBV-negative by routine methods while showing EBNA1 expression in rare tumor cells by RNAscope. The relapse specimens were clonally identical to their onset biopsies, indicating that the lymphoma clone can largely loose the EBV genome over time but traces of EBV infection are still detectable by high-sensitivity methods. We suggest EBV may contribute to lymphoma pathogenesis more widely than currently acknowledged.

## Introduction

About 10–20% of cancers are caused by infectious agents, ranging from 10% of the total number of cancer cases in the most developed parts of western countries and up to 20% in developing countries [1]. Epstein–Barr virus (EBV) is a gammaherpesvirus linked to a number of lymphoid and epithelial malignancies, including Burkitt lymphoma (BL), diffuse large B-cell lymphoma (DLBCL), Hodgkin lymphoma (HL), primary effusion lymphoma (PEL), various types of T/NK-cell lymphomas as well as gastric and nasopharyngeal carcinoma (NPC) [2]. Although the vast majority (>90%) of the world population exhibits antibodies to EBV, the frequency of the virus in EBV-associated tumors is highly variable [3, 4]. EBV is peerless in its ability to turn a normal B lymphocyte with a defined life span into an indefinitely growing immortalized cell. Indeed, after active infection, the virus remains in a latent form in memory B cells that provide the main cellular reservoir of the virus [5, 6]. In addition to a restricted set of viral proteins expressed during latent EBV infection, the virus may impact on host cell homeostasis by interfering with cellular miRNAs expression and by encoding its own miRNAs [7–9]. However, the exact mechanisms by which EBV contributes to lymphoid malignancies are not well known. The role of the virus is further confounded by the fact that, within each EBV-associated cancer, viral infection of tumor cells is documented by routine methods in only a fraction of cases [10–12]. In this regard, a “*hit-and-run*” hypothesis has been proposed. According to this idea, EBV can mediate cellular transformation through an initial “hit” determined by the viral gene expression program. After heritable changes have been induced by the virus in the cellular gene expression and/or after somatic mutations have been acquired in cellular oncogenes/tumor-suppressor genes, the virus genome may not be strictly necessary for tumor maintenance. Then, the virus may be progressively lost from the lymphoma clone (“run”) after each cell cycle, because of the known imperfect duplication and asymmetric partitioning of EBV episomes during S-phase and M-phase respectively [13, 14]. However, so far little data exist to support such hypothesis and routine methods to detect the virus, like immunohistochemistry (IHC) for the viral protein LMP1 and In situ hybridization (ISH) for EBV-encoded RNAs (EBER), may have suboptimal sensitivity [15, 16]. Viral gene and microRNA (miRNA) detection by RT-qPCR in microdissected tumor cells and In situ RNAscope analysis (that enables RNA detection with single-molecule sensitivity in tissues) have been shown to be sensitive and specific tools for pathogens detection [17, 18]. Recently, by applying some of these techniques we were able to document the presence of EBV nucleic acids at low levels in a limited number (*n* = 6/6) of EBER-negative BL cases [19].

Therefore, the aim of the present study was to analyse a larger case series of lymphoma entities, some with an established link to EBV (BL, DLBCL, HL, and follicular lymphoma—FL [20, 21]) and some not (T-lymphoblastic lymphomas (T-LL), hairy cell leukemias (HCL) and mantle cell lymphomas (MCL) by using quantitative PCR and droplets digital PCR (ddPCR) measurement of EBV genes and micro-RNAs in purified neoplastic cells along with highly sensitive RNAscope ISH assay for EBNA1 mRNA, in addition to routine techniques such as immunohistochemistry for LMP1 and EBER-ISH on whole-tissue sections. While IHC for LMP1 and EBER-ISH identified a proportion of EBV-positive tumors overlapping that reported in the literature in BL, DLBCL, FL, and HL, measurement of viral gene load, as well as of viral microRNA and mRNA expression, identified traces of EBV exposure also in occasional tumor cells of samples negative by conventional tools. Since in B cells and epithelial cells EBV epigenetically silences tumor-suppressor genes including *MGMT* and *CDH1*, and since EBV infection in epithelial cancers correlates with promoter hypermethylation of these two genes [22–26], we also analysed the methylation status of such gene promoters in our lymphoma cases. We found that the methylation status of both genes was quite similar in conventional EBV-positive cases and in cases classified as EBV-negative by routine methods but showing traces of EBV infection; in contrast, cases without any footprint of exposure to EBV showed a low level of methylation.

Collectively, our findings provide support to the “*hit-and-run*” hypothesis and may expand the role of EBV in B-cell lymphomagenesis.

## Materials and methods

### Patients

The cases cohort was represented by 148 formalin-fixed and paraffin-embedded (FFPE) samples, retrieved by the Archives of Siena University Hospital, as well as by 19 frozen cHL cases (previously studied for other purposes [27] and 20 fresh HCL cases collected at the Institute of Haematology in Perugia, which were characterized by clinical data, morphology, immunophenotype, cytogenetic and molecular biology consistent with the last World Health Organization criteria for diagnosis [28]. Specifically, we analysed 19 BL, 34 DLBCL, 50 FL, 44 cHL (27 nodular sclerosis, 13 mixed cellularity, 3 lymphocyte-rich, and 1 lymphocyte-depleted), 20 HCL, 10 T-LL, and 10 MCL.

### Cell lines

DNA from six BL cell lines (one EBV-positive: Namalwa; five EBV-negative: Ramos, DG75, BL41, BL2; Akata-2A8), nine cHL cell lines (seven EBV-negative: SUP-HD1, L540, HDLM2, L428, KMH2, L1236, UH-01; two EBV-positive: L591, AM-HLH) and, as controls, two multiple myeloma cell lines (CAG, RPMI) and five acute meloid leukemia cell lines (OCI-AML-3, U-937, KG-1, Kasumi-1, TF-1), was isolated by using Macherey Nagel kit, following manufacturer’s instructions. The amount and quality of DNA were evaluated by measuring the optical density (OD) at 260 nm, the 260/230 and the 260/280 ratios using a Nanodrop spectrophotometer (NanoDrop Technologies LLT, USA).

### Immunohistochemistry and EBER-ISH

Immunohistochemical stainings were performed on all FFPE cases by an automated staining system (Ventana BenchMark ULTRA, Roche diagnostic, Monza-Italy) with appropriate positive and negative controls included in each staining run, as previously described [29, 30]. We used antibodies against EBNA1 (Santa Cruz, Heidelberg, 1EB12) and LMP-1 (Abcam, D24G). ISH for EBER was carried out in each sample on 5 μm-thick section as previously described [29]. A control slide prepared from a paraffin-embedded tissue block containing EBV-positive metastatic nasopharyngeal carcinoma in a lymph node accompanied each hybridization run.

### Laser capture microdissection (LCM)

The neoplastic population of each non-Hodgkin lymphoma sample (including those positive at EBER-ISH) was microdissected from EBER-ISH stained 3 μM-thick FFPE sections. Multiple areas, each one containing ~40–50 cells, were harvested to collect a total number of ~200,000 cells, using a PixCell II laser capture microdissector (Arcturus Engineering, MGW, Florence, Italy). The EBER-staining allowed, in cases with EBER-negative lymphoma cells, to exclude from microdissection even a single EBER-positive small reactive lymphocyte that might impact on the following qPCR analyses. Regarding microdissection of cHL samples, single Hodgkin and Reed/Sternberg (HRS) cells were individually picked (300–500 cells per case from Siena; ~1200–1800 cells per case from Perugia) from 5 µM-thick haematoxylin-eosin stained FFPE sections as described above (for cases from Siena) or from 8 μM-thick haematoxylin/eosin-stained frozen sections as described by Tiacci et al. [27] (for cases from Perugia).

HCL cells were isolated to a purity of ≥95% from patients’ peripheral blood or bone marrow aspirate after red blood cell lysis followed by MACS sorting with CD19-microbeads (Miltenyi Biotec, Bergisch Gladbach, Germany), as previously described [32].

### DNA extraction and processing before PCR

Regarding non-Hodgkin lymphoma cases, the microdissected cells were adhered to a CapSure cap with adhesive transfer film (Arcturus, MWG-Biotech) and then collected into one standard microcentrifuge tube for the DNA extraction by using PicoPure DNA extraction kit Arcturus (Milan, Italy). Regarding FFPE cHL cases from Siena, DNA from 300 to 600 microdissected cells was extracted by the QIAmp DNA Mini Kit (Qiagen Ltd, Crawley, UK) and then subjected to whole-genome amplification (WGA) using the GenomePlex WGA2 kit (Sigma-Aldrich). The amplified DNA (ranging from 2 to 5 mg) was purified using the GenElute PCR clean-up kit (SigmaAldrich) for subsequent PCR amplification. Regarding frozen cHL cases from Perugia, DNA had been extracted and subjected to duplicate WGA as previously described [27]. Full details are given in the Supplementary Materials.

### Quantitative PCR and droplets digital PCR assays to measure EBV Genome Load

All cases were firstly subjected to qPCR assays (Life Technologies Italia, Italy) targeting the BamHI-W and EBNA1 conserved regions of the EBV genome. BamHI-W is a reiterated sequence present at approximately ten copies per EBV genome and it appears to be the most sensitive method to prove the presence of the viral genome, whereas EBNA1 targets a single-copy highly conserved gene essential for maintaining the virus long-term in dividing cells. Human Beta globin (*HBB*) gene was used as reference cellular gene for normalization of the input DNA. According to previous literature [31], a standard curve was generated using tenfold dilutions of Namalwa cell line DNA containing between 500,000 and 0.5 copies of EBV DNA, assuming that diploid Namalwa cell line cells carry two EBV genomes per cell and that each cell contains 6.6 pg of DNA, equivalent to 3.0303 × 10^5^ copies of EBV/μg DNA. Amplification reactions were performed in technical triplicates from 100 ng of test DNA. Each experiment included DNA samples from EBER-positive and EBER- negative cases, as well as water-only controls. Further technical details regarding qPCR and quantification of viral load are given in the Supplementary Materials. Besides technical negative (water) controls for the PCR amplification, as biological negative controls we used 10 samples of T- LL, 20 samples of HCL [32, 33], and 10 samples of MCL, as none of these lymphoma entities have ever been associated to EBV.

Moreover, we independently quantified the absolute copy numbers of EBNA1 and BamHI-W with droplets digital PCR (ddPCR; Biorad) using the same primers and probes, according to the manufacturer’s instructions. Briefly, the PCR reaction was performed using 100 to 150 ng of DNA, 1 × ddPCR Supermix for Probes (BioRad, Hercules, CA, USA), 0.30 µM of each primer, and 0.6 µM of the probe in a total volume of 22 µL. After droplet generation using QX200TM Droplet Generator instrument (BioRad), the generated microdroplets were put into a 96-well plate for amplification. Cycling conditions included preheating at 95 °C for 10 min followed by 40 cycles of denaturation at 94 °C for 30 s, annealing at 58 °C for 60 s, and final heating at 98 °C for 10 min. Then, the PCR plate was transferred to a QX100 droplet reader (BioRad), and fluorescence amplitude data were obtained by QuantaSoft software (BioRad). The absolute copy number of each viral assays was calculated by Bio-Rad software and showed as number of copies/µl. This was converted to absolute copy number per 10,000 cells using the *HBB* absolute copy number/µl and assuming a diploid status for this control cellular gene [34]. More detailed on steps for ddPCR are given in Supplementary Materials.

### Quantitative reverse transcription-PCR assay to measure Viral miRNAs

To quantify the expression of three EBV-encoded miRNAs (EBV-miR-BART9–5p, EBV-miR- BART10-3p, and EBV-miR-BART19-3p), total RNA was extracted from tumor cells microdissected from FFPE lymphoma tissue sections stained for EBER-ISH as described in the previous paragraph on DNA qPCR, with some modifications to preserve RNA (Supplementary Materials). Total RNA (ranging from 160 to 230) ng was extracted from approximately 50,000 non-Hodgkin lymphoma cells and 10 ng of RNA per reaction were reverse transcribed by using TaqMan primers against three EBV-encoded miRNAs, namely EBV-miR-BART9-5p, EBV-miR-BART10-3p, and EBV-miR-BART19-3p (TaqMan assays Cat. # 006884, 004421_mat, 197235_mat) in three separate reactions as described [19]. Then, TaqMan probes specific for each selected viral miRNA were added to analyse by qPCR all cases in technical triplicates. The small cellular RNA RNU6B was used as endogenous control (Applied Biosystems, Applera, Italy) and the expression of each viral miRNAs was calculated using the 2^−Δct^ formula applied to the replicates’ mean. Kruskal–Wallis Test was applied for statistical analysis.

In addition, ddPCR was performed to quantify the absolute copy number of these 3 viral miRNAs, using the same primers and probes as in qPCR [19]. The protocol applied was as follows: 100 ng of RNA, 1 × ddPCR Supermix for Probes (BioRad, Hercules, CA, USA), 2 µl of each primer, and 1 µl of probe in a total volume of 22 µL. 20 µl of each sample were loaded onto middle wells of a cartridges for droplet generation and then transferred into a 96-Well Semi-Skirted PCR plate for amplification. Cycling conditions included preheating at 95 °C for 10 min followed by 40 cycles of denaturation at 94 °C for 30 s, annealing at 58 °C for 60 s, and final heating at 98 °C for 10 min. The absolute copy number of each viral assays was calculated by Bio-Rad software and showed as number of copies/µl. Then, the total number of copies per ddPCR reaction was normalized by that of the housekeeping loading control (RNU6B), and expressed in each case as number of viral miRNA copies/10,000 RNU6B copies as well as percentage of the corresponding average value across conventional EBV-positive cases of the same histology. Full details are given in the Supplementary Materials.

### RNAscope for EBNA1 mRNA

RNAscope is an amplified ISH assay more sensitive than standard ISH for EBV-encoded RNAs to detect viral gene expression. It employs a multiple probe pair design strategy in which two independent probes within each pair (double Z probes) have to hybridize to the target sequence in tandem next to each other for signal amplification to occur, which improves the signal-to-noise ratio. Signal amplification is achieved by a cascade of hybridization of a nucleic acid pre-amplifier followed by multiple amplifiers that serve as a substrate for the subsequent binding of chromogenic molecules to the numerous binding sites in each amplifier [18].

RNA In situ hybridization was performed on FFPE tissue sections or on fixed cytospins of cell lines using the RNAscope 2.0 HD Red Chromogenic Reagent Kit (Advanced Cell Diagnostics, CA) and V-EBV-EBNA1 (Advanced Cell Diagnostic, CA) target probe, according to the manufacturer’s instructions. Each sample was quality-controlled for RNA integrity with a probe specific to the housekeeping *PPIB* mRNA used as positive control. Hybridization signals were visualized by chromogenic reactions using FastRed. Background staining was evaluated using a negative control probe specific for bacterial dihydrodipicolinate reductase (*dapB*); all lymphoma cases analysed did not show any dots for the *dapB* in any cells. Further technical details are given in the Supplementary Materials.

### Methylation studies

Genomic DNA was extracted from five 5-µm-thick whole sections of FFPE non-Hodgkin lymphoma sections using the NucleoSpin Tissue extraction kit (Macherey-Nagel, Germany) according to the manufacturer’s instructions. The amount and quality of DNA were evaluated by measuring the optical density (OD) at 260 nm, the 260/230 and the 260/280 ratios using a Nanodrop spectrophotometer (NanoDrop Technologies LLT, USA). 300 ng of DNA from each sample were used for bisulfite conversion, in which unmethylated cytosine was converted to uracil with the EpiTect Fast DNA bisulfite kit (Qiagen, Hilden, Germany, 59824) according to the manufacturer’s instructions. Human HCT116 DKO Non-methylated and Methylated DNA (Zymo Research, USA, D5014-1/2) were used as standards controls. Full details of bisulfite conversion and subsequent PCR are given in the Supplementary Materials.

## Results

### Findings at conventional immunohistochemistry and In situ hybridization (ISH)

We screened all lymphoma cases for EBV infection by performing standard EBER-ISH. For BL we used 9 EBER-positive cases analyzed in previous papers [17, 19] as positive controls showing the typical nuclear positivity staining for EBNA1 as well as EBER-ISH positivity in almost all neoplastic cells. In addition, we newly collected ten sporadic BL cases for which the EBV status was unknown; of these, only one case was positive at EBER-ISH and immunohistochemistry for EBNA1, while nine BL cases were negative for EBNA1 and EBER. In DLBCL samples, 4 of 34 (12%) samples were positive for EBNA and LMP-1 by immunohistochemistry and for EBER at ISH, whereas the remainder were negative for all three markers. Twelve of 44 (27%) cHL cases were positive for LMP1A and EBER (mixed cellularity, *n* = 5; nodular sclerosis, *n* = 6; lymphocyte-rich, *n* = 1), which were both negative in the other 32 cases. Three of 50 (6%) FL cases were positive for EBER, in keeping with the rare presence of EBV infection in FL [20]. Thus, routine methods detected EBV infection at the expected frequency in our cohort of EBV- associated lymphomas, whereas control lymphomas T-LL (n = 10) and MCL (*n* = 10) were negative at EBER-ISH also as expected.

### Viral genome load by qPCR and droplet digital PCR in primary lymphoma cells

**Fig. 1:**
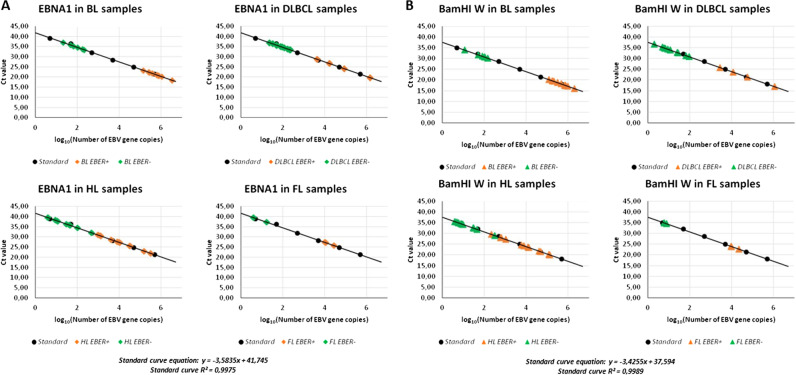
Detection of EBV genome in lymphoma cases by real-time quantitative PCR. Serial dilution of Namalwa DNA containing 500,000 to 0.5 copies of EBV genomes were amplified using primer/probe combinations specific for EBV EBNA1 (**a**) and BamHI-W conserved region (**b**) and the related standard curve was used to quantify the two genes segments in lymphoma cases of the indicated histology. The y-intercept corresponds to Ct values while the x-intercept corresponds to the copy number of each target expressed in log 10 scale.

Quantification by real-time PCR of the EBV genome load in lymphoma cells microdissected from each case (Supplementary Table 1) was extrapolated from the EBNA1 and BamHI-W calibration curves derived from Namalwa cells, where the linear relationship (*R*^2^) reached 0.99 for both genes between the respective Ct value and the log_10_ value of the initial EBV genome copy number (Fig. 1). As expected, qPCR analysis of the 10 EBER-positive BL cases revealed high viral loads in all of them (Fig. 1 and Supplementary Table 1), with an average of 829,744 copies/10,000 cells (ranging from 138,526 to 3,366,413) for EBNA1, and 651,698 copies/10,000 cells (range 122,596–1,991,875) for BamHI-W. High loads of these two genes were documented, as expected, also in EBER-positive cases of DLBCL (n = 4; average of 389,775 EBNA1 copies and 294,316 BamHI-W copies per 10,000 cells), cHL (*n* = 12; average of 51,944 EBNA1 copies and 32,648 BamHI-W copies per 10,000 cells) and FL (*n* = 3; average of 16,679 EBNA1 copies, and 13,854 BamHI-W copies for 10,000 cells). Conversely, and again as expected, the two viral genes were always undetected in all water controls and in all technical triplicates of DNA samples from EBV-unrelated neoplasms (HCL, *n* = 20 cases; T-LL, *n* = 10 cases, MCL, *n* = 10 cases). Interestingly, however, both EBV genes were consistently detected in all technical triplicates of 6/9 EBER-negative BL cases (67%; Fisher’s exact test *p* value < 0.00001 versus 0/40 HCL, T-LL and MCL cases), at low copy numbers ranging between 21 and 210 copies/10,000 cells for EBNA1 and 12–150 copies/10,000 cells for BamHI-W (Fig. 1 and Supplementary Table 1), confirming and extending our previous results in other BL cases [19].

Similarly, 11/30 EBER-negative DLBCL cases were also positive in all technical triplicates (37% of cases, Fisher’s exact test *p* value < 0.0001 versus 0/40 HCL, T-LL, and MCL cases), again at low copy numbers (0–232 EBNA1 copies and 2–95 BamHI-W copies per 10,000 cells; a single case was negative for EBNA1 but positive for five BamHI-W copies/10,000 cells). In the remaining DLBCL cases (19/30), neither EBV gene was detected in any technical triplicates (Fig. 1 and Supplementary Table 1).

In cHL, one or both EBV genes were again detected at low levels in all technical triplicates of 16/32 EBER-negative samples (50%, Fisher’s exact test *p* value < 0.0001 versus 0/40 HCL, T-LL, and MCL cases) with copy numbers per 10,000 cells ranging from 0 to 475 for EBNA1 and from 4 to 315 for BamHI-W (6 cases resulted qPCR-positive for BamHI-W, but not for EBNA1) (Fig. 1 and Supplementary Table 1). cHL cases positive at qPCR were present both in the series from Siena (*n* = 11/16 cases) and in that from Perugia (*n* = 5/16 cases), and included various histologies (nodular sclerosing, *n* = 12; mixed cellularity, *n* = 3; lymphocyte-rich, *n* = 1). Finally, also 3/47 EBER-negative FL cases proved positive in all technical triplicates (6% of cases—Fisher’s exact test *p* value 0.24 versus 0/40 HCL, T-LL and MCL cases), with an average of 4–17 EBNA1 copies and 6–8 BamHI-W copies per 10,000 cells (Fig. 1 and Supplementary Table 1). Neither EBV gene was detected in any technical triplicates of the remaining EBER-negative cHL cases (16/32) and FL cases (44/47).

Thus, we observed consistent presence of EBV genes, at levels widely varying within a low range, in conventionally EBER-negative BL, DLBCL, cHL, and FL cases at notable frequencies (67%, 37%, 50%, and 6%, respectively).

To further confirm these findings and independently measure the absolute copy numbers of EBNA1 and BamHI-W, all EBER-negative samples that resulted qPCR-positive were also analyzed by ddPCR. While both genes were detected at high levels in EBER-positive B-cell lymphoma cases (Supplementary Table 1), ddPCR confirmed the low-level presence of one or both viral genes in all EBER-negative/qPCR-positive samples tested (6/6 BL; 11/11 DLBCL; 16/16 cHL; 3/3 FL), with copy numbers ranging from 1 to 207 copies for BamHI-W/10,000 cells and 0,24 to 302 copies for EBNA1/10,000 cells. Conversely, neither EBV gene was detected in any EBER-negative/qPCR-negative lymphomas studied (3BL, 5 DLBCL, 5 cHL, 5 FL; Supplementary Table 1).

### Viral genome load in BL and HL cell lines by droplet digital PCR

Although the above data were obtained from EBER-negative primary lymphoma cells purified through microdissection, we wanted to further rule out any possible contribution of contaminating reactive cells to the qPCR/ddPCR signal observed in microdissected tumor cells. To this end, we subjected to ddPCR for the EBNA1 and BamHI-W genes several established cell lines of cHL origin (*n* = 9; 7 EBV-negative; two EBV-positive) and BL origin (five EBV-negative; one EBV-positive), using two multiple myeloma and five acute meloid leukemia cell lines as negative controls. As expected, EBV-positive BL and cHL cell lines showed a high copy number in both viral assays (Supplementary Fig. 1). On the other hand, one or both viral genes were detected at low level in 6/7 EBV-negative HL cell lines (copy number ranging between 1.3 and 11.4 copies/10,000 cells for BamHI-W and 0.3–2.6/10,000 cells for EBNA1) and 4/5 EBV-negative BL cell lines (copy number ranging between 0.4 and 2.8 copies/10,000 cells for BamHI-W and 0.11–1.7/10,000 cells for EBNA1). Conversely, neither EBV gene was detected in any cell lines used as negative controls (Supplementary Fig. 1).

### In situ detection of EBNA1 mRNA expression

**Fig. 2:**
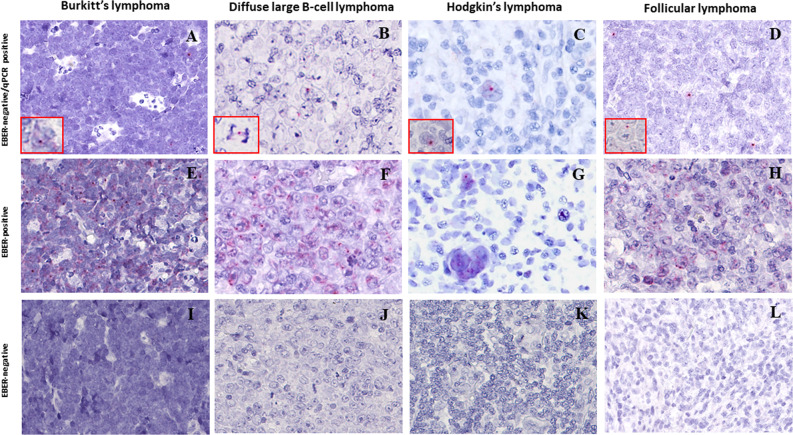
Sensitive *In situ* detection of EBV by RNAscope analysis in lymphoma biopsies. (A, E, I) BL; (B, F, J) DLBCL; (C, G, K) cHL; (D, H, L) FL. A red punctuate staining produced by EBNA1 mRNA molecules is detected in neoplastic cells, often at the nuclear periphery. Single red dots were detected in occasional lymphoma cells of EBER-negative cases positive for EBNA1 at qPCR (**a**–**d**), compared with often multiple intracellular dots in most lymphoma cells of EBER-positive controls (**e**–**h**), and to no signal whatsoever in lymphoma cells of cases negative at both EBER-ISH and EBNA1-qPCR (**i**–**k**). Original magnification: 25×. Insets in A-D show single EBNA1-positive lymphoma cells (magnified in **a** or undergoing mitosis in **b**).

**Fig. 3:**
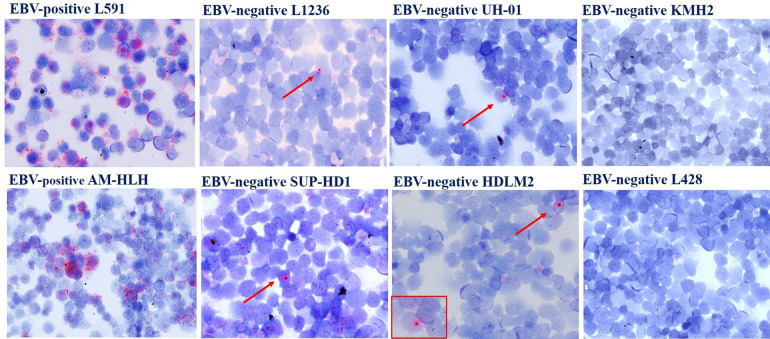
Sensitive In situ detection of EBV by RNAscope analysis in cHL cell lines. Single dots of EBNA1 mRNA were detected in rare lymphoma cells (pointed by the arrow) of 4 conventional EBV-negative cell lines scoring positive on ddPCR analysis for EBNA1 (L1236, UHO1, SUP-HD1, HDLM2), but in no cells of 2 conventional EBV-negative cell lines lacking EBNA1 signal on ddPCR (KMH2, L428). In contrast, conventional EBV-positive cHL cell lines used as positive controls (L591, AM-HLH) showed a higher number of RNAscope dots per cell in most cells.

To provide an In situ validation of the above findings, we checked EBNA1 mRNA expression by sensitive RNAscope that, by preserving the morphological context, allowed us to identify the morphological nature of cells contributing to the positive qPCR/ddPCR signals. We analyzed 32 and 20 EBER-negative cases that respectively did or did not show a signal at DNA qPCR for EBNA1 (Supplementary Table 1). The characteristic red punctuate nuclear staining pattern of such assay was observed, usually as a single dot and occasionally during mitotic division, in scattered rare cells of morphologically clear tumor origin in most EBER-negative/EBNA1-qPCR-positive lymphoma cases tested (6/9 BL, 8/10 DLBCL, 7/10 cHL, 2/3 FL), but in no tumor cells of any EBER- negative/EBNA1-qPCR-negative cases (2 BL, 5 DLBCL, 8 cHL, 5 FL) (Fig. 2; Supplementary Table 1) that were instead positive for the control *PPIB* mRNA probe (not shown). The lymphoma cases with tumor cells positive for EBNA1 at the DNA level by qPCR but not at the RNA level by RNAscope (i.e., 3/6 BL, 2/10 DLBCL, 3/10 cHL, 1/3 FL) were consistently those with the lowest EBNA1 gene load at DNA qPCR within each lymphoma entity (Supplementary Table 1). Conversely, in all EBER-positive lymphoma cases tested (10/10 BL, 4/4 DLBCL, 12/12 cHL, and 3/3 FL) most neoplastic cells were stained as expected, and they often showed multiple red dots per nucleus (Fig. 2 and Supplementary Table 1), indicating a higher viral genome load per cell. Negative controls (10 T-LL and 10 MCL cases) did not show any signal regarding EBNA1, while showing positivity for the control gene, as expected (not shown). Similarly, RNAscope analysis of cultured cHL cells cytospun on glass slides revealed multiple EBNA1 red dots in most cells of conventional EBV-positive lines and a single dot in rare cells of all 4 conventional EBV-negative lines that showed traces of EBNA1 infection on ddPCR; conversely, not a single EBNA1 dot was observed in any cells of a conventional EBV-cell line (KMH2) that showed a ddPCR signal for BamHI-W but not for EBNA1, attesting to the high specificity of the RNAscope assay (Fig. 3).

Thus, we confirmed the presence of EBV genes and their transcription in neoplastic cells of lymphoma cases and cell lines otherwise classified as EBV negative by conventional methods. We also tried to microdissect such rare lymphoma cells positive for EBNA1 at RNAscope in order to further confirm their belonging to the neoplastic clone through the identification of the same immunoblobulin gene rearrangement; however, this turned out to be not technically feasible as the often single RNAscope signal observed in the rare positive cells under the optimal optical conditions of a diagnostic microscope was impossible to clearly appreciate at the inverted LCM microscope in tissue sections without coverslip (as required by the microdissection procedure).

**Fig. 4:**
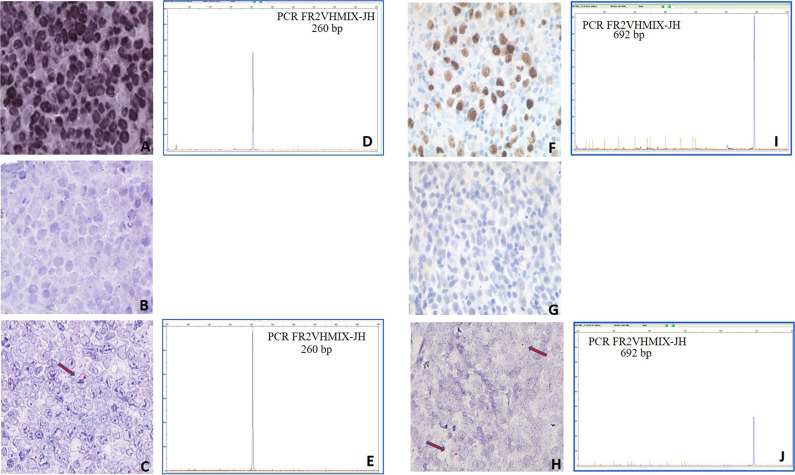
RNAscope and clonality analyses of a DLBCL case showing distinct tumor areas with discordant EBV status (a–e) and an EBV-positive DLBCL case relapsed as EBV-negative by conventional methods (f–j). DLBCL case characterized by an area of strongly EBER-ISH positive cells (**a**) and a region of completely negative EBER-ISH cells (**b**) that however showed occasional EBNA1 mRNA signals by RNAscope (red chromogen pointed by the arrow; panel **c**). An identically sized clonal immunoglobulin heavy chain variable gene rearrangement (260 base pairs) was identified in both the EBER-positive (**d**) and EBER-negative (**e**) microdissected areas. DLBCL case initially EBER-positive (brown chromogen; **f**) and relapsed as negative by EBER-ISH (**g**) while showing rare EBNA1 mRN signals by RNAscope (red chromogen; arrows in **h)**. An identically sized abnormal clonal immunoglobulin heavy chain variable gene rearrangement (692 base pairs) was identified in both the EBER-positive (**i**) and EBER-negative (**j**) biopsies after microdissection of tumor cells.

**Fig. 5:**
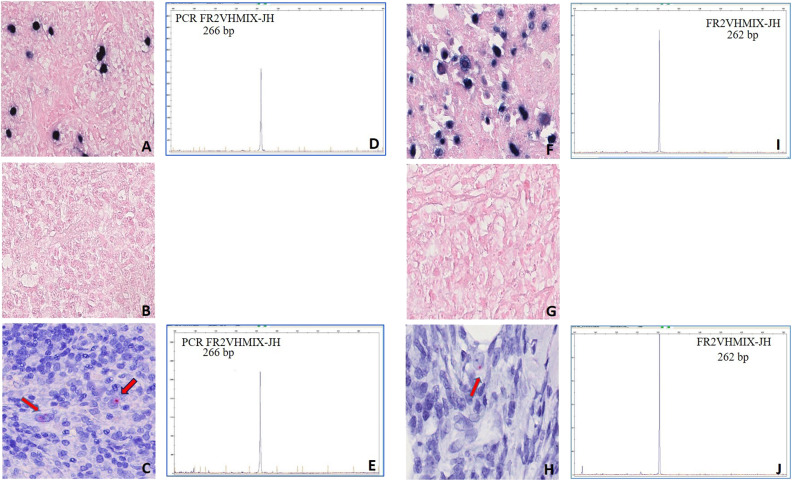
RNAscope and clonality analyses of an EBV-positive cHL case relapsed as EBV-negative by conventional methods (a–e) and an EBV-positive cHL case evolved to a lymphoma intermediate between cHL and DLBCL EBV-negative by conventional methods (f–j). Clonal EBV infection was detected by EBER-ISH in the onset biopsy of a cHL case (blue chromogen; **a**). Conversely, EBER-ISH staining resulted negative in the relapse biopsy post-therapy (**b**). However, occasional red dots indicating EBNA1 mRNA were observed in rare tumor cells of the relapse biopsies (panel **c**). An identically sized clonal immunoglobulin heavy chain variable gene rearrangement (266 base pairs) was identified in HRS cells microdissected from both the initial (panel **d**) and the relapse (**e**) biopsies. EBER-ISH positive cHL (blue chromogen; **f**) evolved in EBER-ISH-negative lymphoma with features intermediate between cHL and DLBCL (**g**). Occasional red dots indicating EBNA1 mRNA were observed in rare tumor cells of the relapse biopsies (**h**). An identically sized clonal immunoglobulin heavy chain variable gene rearrangement (262 base pairs) was identified in whole-tissue sections from both the initial (**i**) and the relapse (**j**) biopsies.

We then studied four additional interesting B-cell lymphoma cases: one DLBCL, characterized by an area of strongly EBER-positive cells separated from another completely negative (Fig. 4a–e); one DLBCL, originally EBER-positive but relapsed 2 years after therapy as EBER-negative (Fig. 4f–j); one cHL, initially EBER-positive and then evolved to an EBER-negative lymphoma with features intermediate between DLBCL and cHL (Fig. 5a–e) 1 year after therapy; and one EBER-positive cHL that relapsed 3 years after therapy as EBER-negative (Fig. 5f–j). We, therefore, show that EBV loss at relapse, which had been already documented in cHL [35, 36], can occur also in DLBCL. We next looked for potential EBV vestiges in the three relapse biopsies and in the EBER-negative area of the otherwise EBER-positive DLBCL biopsy, by performing RNAscope for EBNA1. We indeed observed in all four cases single red dots in scattered tumor cells in the relapse biopsies or in the EBER-negative area (Figs. 4, 5) compared with often multiple red signals in most tumor cells of the matched onset biopsies and of the EBER-positive area, respectively (not shown). We also confirmed the clonal relation between the onset and relapse samples, as well as between the EBER-positive and EBER- negative areas of the same DLBCL biopsy, by fragment length analysis of the immunoglobulin heavy chain variable gene rearrangement following microdissection of ~1500–2000 single HRS cells and of groups of ~40–50 non-Hodgkin lymphoma cells from hematoxylin/eosin-stained sections (Figs. 4, 5).

Finally, we addressed whether or not the weak EBNA1 signal observed on RNAscope in rare tumor cells of conventional EBV-positive lymphomas could be due to secondary infection by a bystander EBV-infected cell undergoing viral lytic replication. To do so, we investigated In situ the expression pattern that EBNA1 displays in normal latently EBV-infected lymphoid cells that are occasionally detected by EBER-ISH staining in reactive lymph nodes or in the reactive component of lymphoma tissues (especially cHL). In 2 such biopsies with readily detectable EBER-positive reactive lymphocytes (*n* = 1 reactive lymphadenitits; *n* = 1 cHL—Supplementary Fig. 2), RNAscope for EBNA1 consistently produced an intense staining (homogeneous or comprising multiple dots) in small reactive lymphoid cells (presumably in EBV latency I, i.e., expressing not only EBER but also EBNA1 [5, 6, 37]) or in activated germinal center B cells (presumably in latency II, i.e., expressing not only EBER and EBNA1 but also LMP1 [38]. This strong staining pattern resembled that of tumor cells in EBER-positive lymphomas much more than the weak staining (often consisting of a single dot) observed in rare tumor cells of EBER-negative cases and lines scoring positive at qPCR/dPCR for EBNA1 (Figs. 2–5). Because such intense EBNA1 staining likely typifies EBV infection (in its various latent forms) of both normal and tumor lymphoid cells, secondary EBV infection of rare tumor cells within an originally EBV-negative lymphoma clone is unlikely to explain the EBNA1 expression pattern actually shown by those rare lymphoma cells, reinforcing the alternative explanation of an originally clonal EBV infection followed by progressive loss of the viral genome from most tumor cells and persisting traces of it in only a few.

Altogether, these findings further support the hypothesis that even if the lymphoma clone can largely loose the EBV genome over time, its traces can still be detected by high-sensitivity methods.

### EBV-encoded microRNAs expression by qPCR and droplet digital PCR

**Fig. 6:**
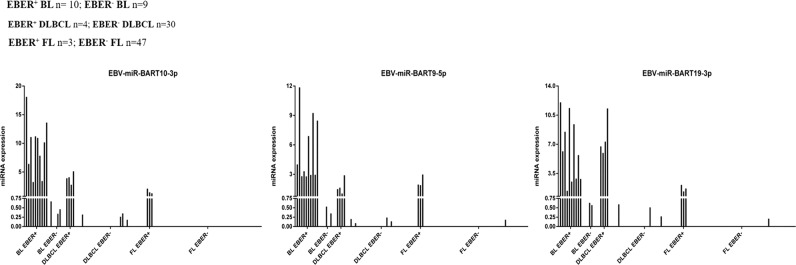
Expression of EBV-encoded miRNAs in EBER-negative cases by RT-qPCR. Expression values (2^−Δct^) are reported on the *y*-axis, indicating low-level viral miRNAs expression in several samples classified as EBER-negative (red bars), compared with higher expression in all EBER-positive cases (blue bars).

Based on our previous findings in BL [19], as an additional validation we analysed the expression of three EBV-encoded miRNAs, namely EBV-miR-BART9-5p, EBV-miR-BART10-3p, and EBV- miR-BART19-3p in non-Hodgkin lymphoma cells microdissected from EBER-stained tissue sections. EBER-positive samples showed a clear expression of viral miRNAs, but also most of the tested EBER-negative cases that were positive at DNA qPCR for EBNA1 presented some degree of expression of at least one EBV-encoded miRNA in all technical triplicates performed (6/9 BL, 7/11 DLBCL, 1/3 FL) (Fig. 6 and Supplementary Table 1).

By ddPCR we further confirmed, in all cases classified as EBER-negative but resulted qPCR-positive for miRNAs, the presence of viral miRNA copies ranging from 1 to 9% of conventional EBV-positive lymphomas (Supplementary Table 1). Conversely, no ddPCR signals were ever detected in any technical triplicate of any tested EBER-negative lymphoma (19 DLBCL, 44 FL) that were negative at qPCR for EBV miRNAs.

### Exome-wide somatic mutation load in relation to EBV status

**Fig. 7:**
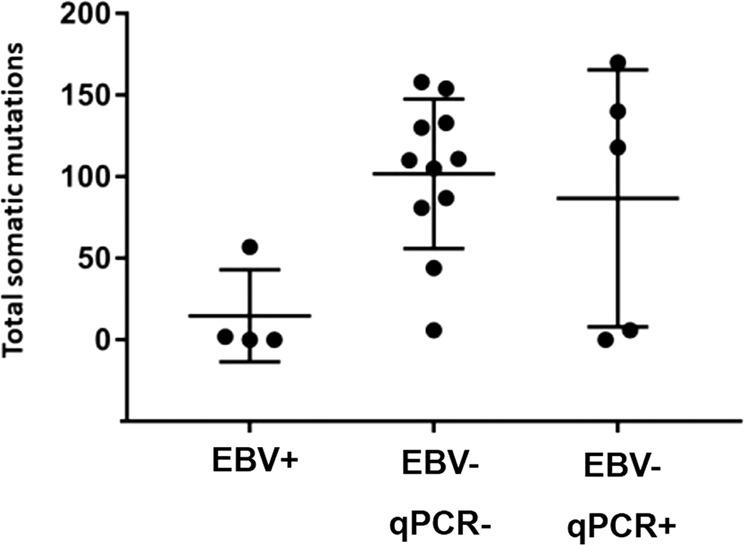
Exome-wide somatic mutation load in relation to EBV status. Load of total somatic mutations (mean ± SD as error bar) present in a major lymphoma clone of cHL cases (circles) EBER-positive (*n* = 4, denoted as EBV-positive), EBER-negative/qPCR-negative (*n* = 11, denoted as EBV-negative qPCR-negative) and EBER-negative/qPCR-positive (*n* = 5, denoted as EBV-negative qPCR-positive). The same result was obtained when considering only somatic nonsynonymous mutations (not shown).

In a previous whole-exome sequencing study of tumor and normal cells microdissected from cHL biopsies, the load of total somatic mutations (whether nonsynonymous or silent/non-coding) that were present in a major tumor clone was much lower in EBER-positive lymphoma cells (*n* = 4 cases; median 0 mutations, range 0–64) than in EBER-negative ones (*n* = 30 cases; median 118 mutations, range 0–665; *p* value < 0.01) [27], suggesting that persistent viral gene expression during lymphomagenesis may relieve the pressure toward selection of exome-wide mutagenic mechanisms. Tumor cells of 16/30 EBER-negative cHL cases previously studied [27] could be assessed by qPCR for EBV genes in the present study, with 11 turning out to be negative and 5 positive (Supplementary Table 1). Interestingly, the somatic mutation load of the EBER- negative/qPCR-positive subset appeared relatively similar to EBER-negative cases, with 3/5 cases showing a conspicuous number of mutations (Fig. 7); these 3 cases all carried specific gene mutations (respectively in *STAT3*, *SOCS1*, *TP53*; in *MYD88*; and in *CD95/FAS*) that activate signaling pathways (JAK-STAT and NF-kB) or inactivate cellular processes (apoptosis) known to be respectively induced and evaded by EBV in cHL [27].

### Methylation assay findings

**Fig. 8:**
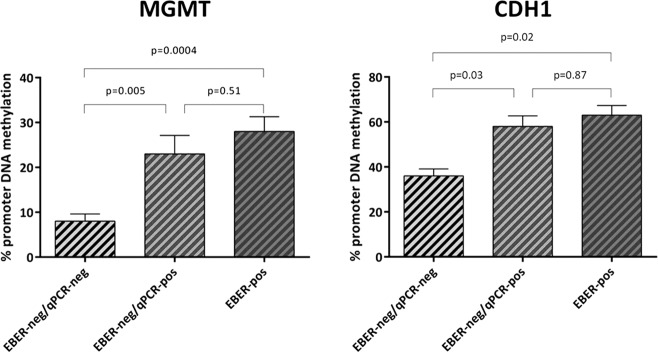
Methylation assay findings. The methylation level of *MGMT* and *CDH1* promoter in 17 non-Hodgkin lymphoma EBER-positive cases overlapped that detected in 20 EBER- negative/qPCR-positive cases, whereas it was higher than observed in EBER-negative/qPCR-negative cases (*n* = 20). *P* values were calculated according to *T*-test.

The promoter region of some tumor-suppressor genes, including *CDH1* and *MGMT*, has been shown to become hyper-methylated during EBV-induced primary B-cell transformation [23, 24]. Therefore, considering that these heritable alterations could be maintained also after the loss of the virus, we investigated the methylation pattern of the *CDH1* and *MGMT* gene promoters in whole lymphoma tissue sections. We compared the methylation status of the 17 EBER-positive cases (10 BL, 4 DLBCL, 3 FL) to that observed in cases classified as EBER-negative but in which we detected the presence of the virus by DNA qPCR (*n* = 20; 6 BL, 8 DLBCL, 6 FL). Interestingly, the methylation status of these two groups was quite similar (28 and 23% respectively for *MGMT*; 58 and 63% respectively for *CDH1*) (Fig. 8). In contrast, EBER- negative/qPCR-negative cases (*n* = 20; 10 DLBCL, 10 FL) showed a lower level of promoter methylation in comparison to both EBER-positive cases and EBER-negative/qPCR-positive cases (Fig. 8).

## Discussion

Epstein–Barr virus plays a pathogenetic role in several lymphoid and epithelial malignancies [2, 39, 40], and the methods generally used to determine EBV status of a cancer are represented by in-situ hybridization for EBER and immunohistochemistry for LMP1 or EBNA1. By these techniques, the proportion of cases clonally infected by EBV is >95% in endemic BL, 10–30% in sporadic BL and cHL of western countries, and 1–10% in DLBCL. Whereas the pathogenetic link with EBV is easy to conceive in tumor cases clonally expressing one or more viral products, it has been proposed that the virus might be implicated also in virus-negative tumors through a “*hit-and-run*” mechanism.

This theory postulates that the transforming events initially provided by the virus are later functionally replaced by stable (epi)genetic changes of the host cell. Then, the viral episomal genome, whose propagation during cell proliferation is intrinsically imperfect, may be progressively lost from the tumor clone as it does not provide anymore a fitness advantage, and can actually be even counterproductive being a potential target of the host immune response [13, 14, 19].

Although conventional EBV-positive BL (whether endemic or sporadic) and cHL do harbor fewer driver gene mutations compared with their EBV-negative counterparts [27, 41], the hypothesis of “*hit- and-run*” oncogenesis is difficult to formally proof. The previous few historical attempts to directly address it in primary cases of EBV-associated lymphomas (BL [42] and cHL [43–45]) led to controversial results. This is particularly the case for cHL where a further major technical challenge is represented by the rarity of tumor cells (typically <5%) in the involved tissue and where opposite results were reported in pediatric [43] versus adult [44, 45] series, respectively supporting and refuting the “*hit-and-run*” hypothesis. More recently, by adopting a methodological strategy combining optimal sensitivity (qPCR for EBV nucleic acids on pure lymphoma cell populations isolated through microdissection of conventional EBV-negative sporadic BL biopsies) and high specificity (EBER staining of tissue sections to avoid co-microdissection of even a single normal EBER-positive cell), we observed traces of EBV infection in 6/6 of conventional EBV-negative BL [19].

In the present study, we analysed a larger and more comprehensive series of typical EBV-associated lymphomas, including DLBCL and adult cHL in addition to BL. This was performed by applying several methodological approaches rigorously guarded not only against technical artifacts by means of several water controls, but also against false positives of non-technical origin by means of biological controls consisting of several EBV-unrelated neoplasms (20 HCL, 10 T-LL, and 10 MCL). All these quantitative analyses documented the consistence presence at a low level of one or more of the 5 EBV nucleic acids tested (DNA of BamHI-W and EBNA1; and the miRNAs BART8- 5p, BART10-3p, and BART19-3p) in tumor cells of several B-cell lymphomas classified as EBV-negative by routine methods; these included 37% (11/30) DLBCL, 50% (16/32) cHL and, strikingly, 80% BL (12/15 cases, considering also those previously reported in [19]). This proportion was considerably lower in FL (6%; 3/47 cases), for which the pathogenetic link with EBV is more elusive as only 2.6% of FL cases have been reported to be EBV-positive by standard methods [20]. In contrast, none of the 40 biological negative controls showed any positive signal for the EBV nucleic acids tested (BamHI-W and EBNA1). Moreover, although it can be theoretically hypothesized that the qPCR signal detected in EBER-negative DLBCL, BL and FL cases might have originated from the co-microdissection of a few normal occult EBER-positive cells even if extreme care was taken to isolate areas of tumor cells completely devoid of any EBER staining, the same argument is not likely to hold for cHL cases, where the normal cells surrounding the single microdissected HRS cells (and thus potentially contaminating the microdissection procedure) are known to be mostly T cells not infected by EBV. Another argument against contamination is the fact that, among EBER-negative cases, the highest frequency of EBV-positivity at qPCR was observed in BL despite this being the lymphoma entity with the lowest non-tumor content in tissue biopsies.

Most importantly, by using the highly sensitive RNAscope analysis, a ISH technology enabling single-molecule detection and preservation of tissue morphology, we orthogonally validated In situ the presence of EBNA1 mRNA in occasional morphologically neoplastic cells of lymphoma cases otherwise classified as EBV negative by conventional methods, at an expression level likely lower (often a single red dot per cell) than conventional EBV-infected tumor cells (often showing multiple red dots per cell).

Although it was technically impossible to microdissect these rare lymphoma cells EBV-positive only at RNAscope for further proving by immunoglobulin gene sequencing the clonal relationship with their EBV-negative counterparts, we note that the morphology of such cells was always neoplastic both in the non-Hodgkin lymphoma cases (medium to large size, more cytoplasm, finer chromatin with a nucleolus often present) and in cHL (typical HRS cell morphology). Therefore, although normal EBV-infected cells can assume diverse morphologies (including activated ones, such as immunoblastic or HRS-cell like) in addition to the more frequent one of a resting lymphocyte, the fact that none of the cells EBV-positive only at RNAscope ever displayed the morphology of a resting lymphocyte, and that they all displayed a stereotypical tumoral morphology instead, argues against the normal nature of these RNAscope-positive cells. Finally, the neoplastic nature of those primary cells weakly expressing EBNA1 was corroborated by ddPCR and RNAscope analyses formally demonstrating, in unequivocal tumor cells of conventional EBV-negative cHL and BL cell lines, the presence and expression of EBV genes with a pattern resembling that of conventional EBV-negative primary lymphomas positive at qPCR, ddPCR, and RNAscope.

Traces of the previously clonal EBV infection were also detected by RNAscope in scattered tumor cells of 4 B-cell lymphoma cases (1 DLBCL and 2 cHL) that relapsed as EBER- ISH negative while being positive at disease onset, as well as in an EBER-negative area separated, within the same DLBCL biopsy, from an EBER-positive area; immunoglobulin rearrangement analysis confirmed a clonal relation between the lymphoma at onset (EBER-positive) and relapse (EBER-negative), as well as between the two distinct area of the same DLBCL biopsy. Therefore, although we cannot formally exclude that the RNAscope signal occasionally detected in the other EBER-negative lymphomas we analyzed (Supplementary Table 1 and Fig. 1) may result from secondary EBV infection of rare lymphoma cells, the above described DLBCL and cHL cases losing EBV at relapse (or losing it even in the same biopsy), support loss of the viral genome from a tumor that initially was clonally infected as a plausible origin also of the RNAscope signal seen in the other EBER-negative lymphomas analyzed. We also note that this RNAscope staining often consisted in a single dot per cell in contrast to the more abundant EBNA1 labeling of clonally infected EBER-positive lymphoma cells and of normal lymphoid cells latently infected by EBV that can be occasionally observed in tissue biopsies. Such a different staining pattern is an additional argument pointing to loss of viral genomes as a more likely scenario than secondary EBV infection of rare tumor cells, as the latter should result in a higher intracellular viral genome load like that we observed in the context of primary infection of normal or neoplastic B cells. Loss of the EBV genome may therefore also explain the significant minority of EBV-infected B-cell lymphoma cases showing EBER positivity in only a fraction of cells (10/27 cases in [53, 54]).

Progressive loss of viral episomes after cellular divisions [14, 46] within a lymphoma clone that in origin was clonally infected is a plausible mechanism underlying the results we observed in EBER-negative lymphomas showing traces of EBV infection. However, we cannot exclude, especially in cell lines continuously growing in vitro since decades, that the RNAscope signal seen in rare tumor cells of EBER-negative lymphomas is contributed by viral DNA integration into the cellular genome; indeed, evidences suggest that the EBV genome can randomly integrate at vulnerable sites of the cellular genome resulting in host genome instability or deregulated gene expression [47–52].

The results obtained with these analyses directly assessing the presence of the virus in conventional EBV-negative primary tumors and cell lines suggest that EBV infection might have happened in the early pathogenesis of a significantly greater proportion of B-cell lymphoma cases than commonly thought, in keeping with the “*hit-and-run*” hypothesis. Furthermore, indirect correlative analyses on genetic and epigenetic changes potentially associated to EBV infection are also compatible with the “*hit-and-run*” hypothesis. Indeed, the exome-wide somatic mutation burden was low in conventional EBV-positive cHL cases, while being higher in conventional EBV-negative cases and relatively similar in the group with traces of EBV infection and in the group without. However, we acknowledge the limited size of the group of lymphoma cases with traces of EBV infection and with available whole-exome sequencing data, such that these preliminary findings need confirmation in a larger case series.

Likewise, at the epigenetic level, the *CHD1* and *MGMT* gene promoters (which become hypermethylated during EBV-induced primary B-cell transformation) [22–26] exhibited, in lymphoma cases of diverse histologies (BL, DLBCL, and FL) harboring only traces of EBV infection, a considerable level of methylation that was similar to that observed in conventional EBV-positive cases and greater than in cases not featuring any traces of EBV infection, These findings extend to, and refine within, B-cell lymphomas the association between EBV infection and *CDH1/MGMT* promoter methylation previously described in epithelial cancers [25].

Our results do not affect the diagnostic work-up as conventional EBV-positive lymphomas should still be diagnosed accordingly to the current WHO criteria [11]; however, whether lymphoma cases showing only traces of EBV infection have some specific biological and/or clinical features may warrant further study. Also, although in our relatively small series of adult BL the majority of EBER-negative cases showed traces of EBV infection (this paper and ref. [19]), we are aware that BL often develops in EBV- seronegative children presumably not yet infected by EBV [55] and we do not claim that Burkitt lymphomagenesis requires EBV infection of tumor cells in each and every pediatric or adult case.

In conclusion, collectively, our findings expand the spectrum of B-cell lymphoma cases potentially linked with EBV infection and may support the efforts toward the design and development of prophylactic vaccination strategies against this virus for reducing the burden of EBV-associated disease worldwide [56].

## Supplementary information

Supplementary Figure 1

Supplementary Figure 2

Supplementary Table 1

Supplementary Materials
