# Correction: Frequent traces of EBV infection in Hodgkin and non-Hodgkin lymphomas classified as EBV-negative by routine methods: expanding the landscape of EBV-related lymphomas

**DOI:** 10.1038/s41379-020-0608-y

**Published:** 2020-06-29

**Authors:** Lucia Mundo, Leonardo Del Porro, Massimo Granai, Maria Chiara Siciliano, Virginia Mancini, Raffaella Santi, Lynnette Marcar, Katerina Vrzalikova, Federica Vergoni, Gioia Di Stefano, Gianluca Schiavoni, Giovanna Segreto, Noel Onyango, Joshua Akelo Nyagol, Teresa Amato, Cristiana Bellan, Ioannis Anagnostopoulos, Brunangelo Falini, Lorenzo Leoncini, Enrico Tiacci, Stefano Lazzi

**Affiliations:** 1 Section of Pathology, Department of Medical Biotechnology, University of Siena, Siena, Italy; 2 Section of Pathology, University of Florence, Florence, Italy; 3 BioMaterials Cluster, Bernal Institute, University of Limerick, Limerick, Ireland; 4 Ireland, Health Research Institute (HRI), University of Limerick, Limerick, Ireland; 5 Institute of immunology and Immunotherapy, University of Birmingham, Birmingham, UK; 6 Section of Haematology and Clinical Immunology, Department of Medicine, University and Hospital of Perugia, Perugia, Italy; 7 Department of Clinical Medicine and Therapeutics, Unit of Medical Oncology, University of Nairobi, Nairobi, Kenya; 8 Department of Anatomical Pathology, University of Charité Berlin, Berlin, Germany

**Keywords:** Molecular biology, Tumour virus infections

Correction to: *Modern Pathology*

10.1038/s41379-020-0575-3

The article *Frequent traces of EBV infection in Hodgkin and non-Hodgkin lymphomas classified as EBV-negative by routine methods: expanding the landscape of EBV-related lymphomas* was originally published electronically on the publisher’s internet portal on 1st June 2020 without open access. With the authors’ decision to opt for Open Choice the copyright of the article changed to © The Author(s) 2020 and the article is forthwith distributed under a Creative Commons Attribution 4.0 International License (https://creativecommons.org/licenses/by/4.0/), which permits use, sharing, adaptation, distribution and reproduction in any medium or format, as long as you give appropriate credit to the original author(s) and the source, provide a link to the Creative Commons licence, and indicate if changes were made.

