# Supplementary figures and images for: Frequent traces of EBV infection in Hodgkin and non-Hodgkin lymphomas classified as EBV-negative by routine methods: expanding the landscape of EBV-related lymphomas
Source: Mod Pathol. 2020 Jun 1;33(12):2407–21. doi: 10.1038/s41379-020-0575-3 (PMC7685982; doi:10.1038/s41379-020-0575-3)

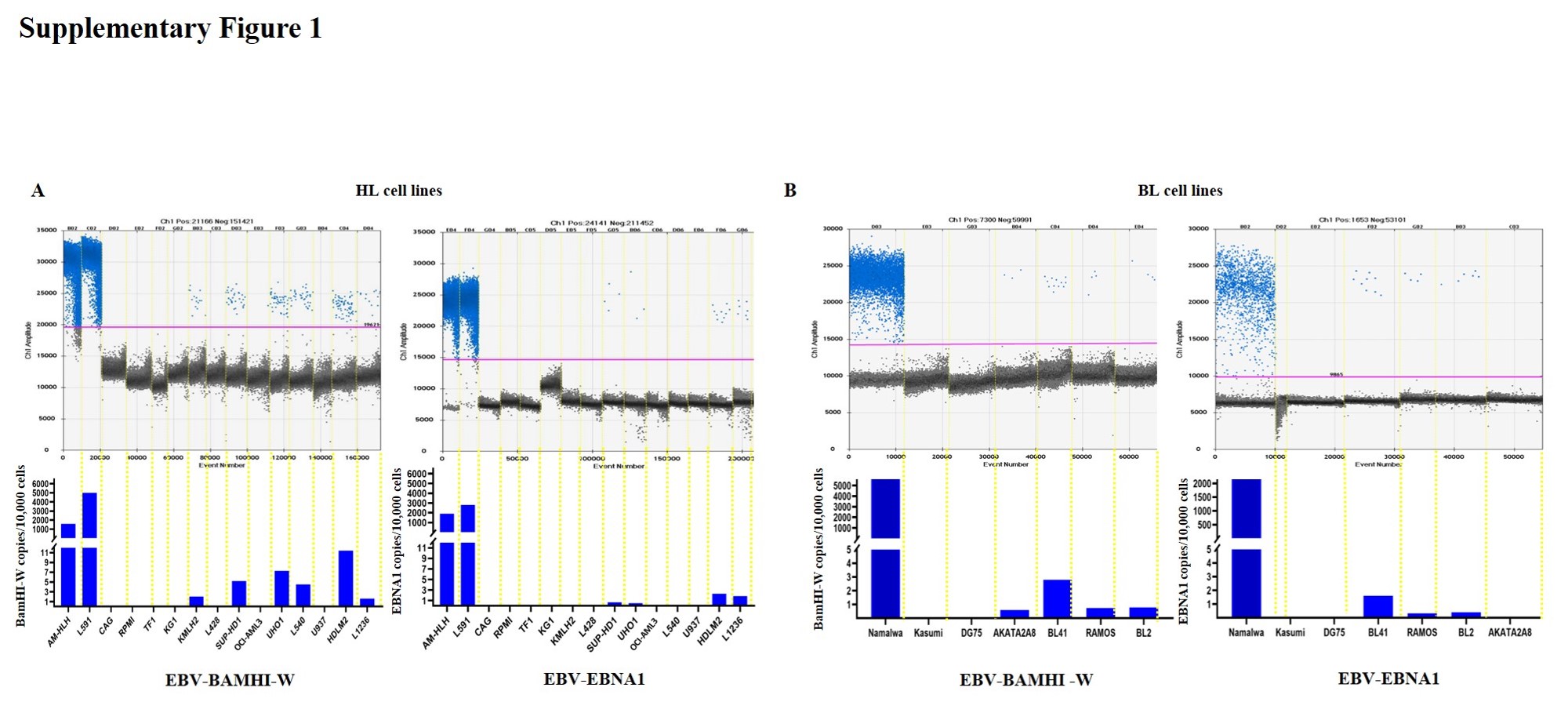

Supplement: Supplementary file 1 — Supplementary Figure 1 [file 41379_2020_575_MOESM1_ESM.jpg]

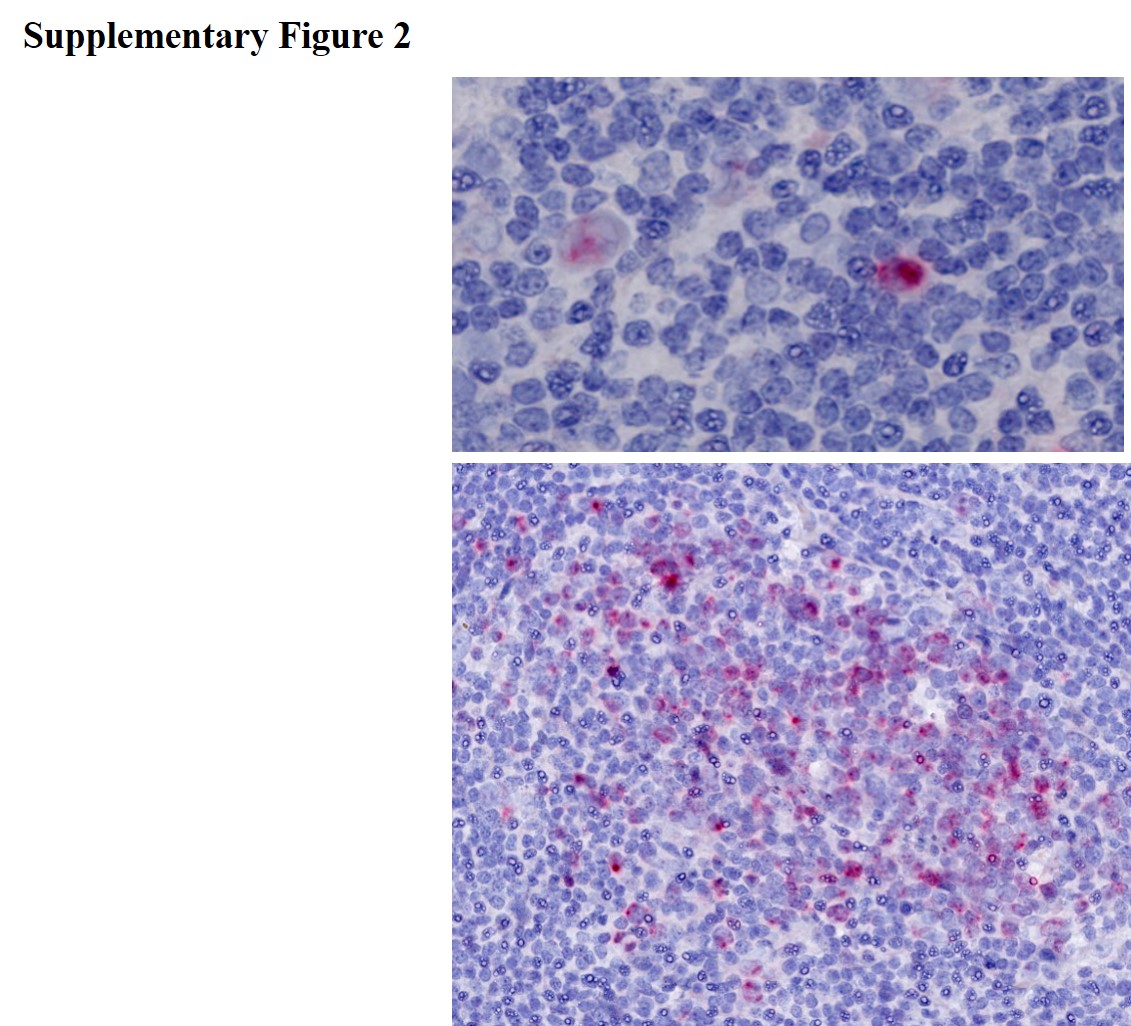

Supplement: Supplementary file 2 — Supplementary Figure 2 [file 41379_2020_575_MOESM2_ESM.jpg]
