# Supplementary material for: Frequent traces of EBV infection in Hodgkin and non-Hodgkin lymphomas classified as EBV-negative by routine methods: expanding the landscape of EBV-related lymphomas
Source: Mod Pathol. 2020 Jun 1;33(12):2407–21. doi: 10.1038/s41379-020-0575-3 (PMC7685982; doi:10.1038/s41379-020-0575-3)
Supplement: Supplementary file 4 — Supplementary Materials [file 41379_2020_575_MOESM4_ESM.doc]

SUPPLEMENTARY MATERIALS AND METHODS

**Immunohistochemistry and EBER-ISH**

Immunohistochemical stainings were performed on all FFPE cases by an automated staining system Ventana BenchMark ULTRA, Roche diagnostic, Monza-Italy) with appropriate positive and negative controls included in each staining run. No epitope retrieval was exploited. UltraView Universal Detection Kit (Ventana) using HRP multimer and DAB (as chromogen) was employed. ISH for EBER was carried out in each sample on 5 μm-thick section as previously described (1). A control slide prepared from a paraffin-embedded tissue block containing EBV-positive metastatic nasopharyngeal carcinoma in a lymph node accompanied each hybridization run.

**DNA Extraction and Processing before PCR**

Regarding non-Hodgkin lymphoma cases, the microdissected cells were adhered to a CapSure cap with adhesive transfer film (Arcturus, MWG-Biotech) and then collected and placed directly onto one standard microcentrifuge tube containing 10 µl of extraction solution (Buffer and Proteinase K, Arcturus). The tube, which had been preheated upright in a 37°C oven for 5 minutes, was placed upside down so that the digestion buffer contacted the tissue on the cap. The tube was then incubated overnight at 60°C and centrifuged for 5 minutes. The reaction was heated to 95°C for 8 minutes to inactivate the Proteinase K, DNA was extracted using PicoPure DNA extraction kit- Arcturus (Milan, Italy) and quantified by Nanodrop spectrophotometer (NanoDrop Technologies LLC, USA) yielding 1.2-1.6 µg of DNA per case before PCR amplification.

Regarding FFPE cHL cases from Siena, a CapSure cap with adhesive transfer film containing groups of 300-600 cells was placed into a pre-heated standard microcentifuge tube, which had been filled with 10 µl of extraction buffer and was then placed upside down as described above for 16 hours, followed by centrifugation to collect the content on the bottom of the tube. DNA was then extracted by the QIAmp DNA Mini Kit (Qiagen Ltd, Crawley, UK) and subjected to whole-genome amplification (WGA) using the GenomePlex WGA2 kit (Sigma-Aldrich). The amplified DNA (ranging from 2 to 5 micrograms) was purified using the GenElute PCR clean-up kit (Sigma- Aldrich) for subsequent PCR amplification. Regarding frozen cHL cases from Perugia, which had been already processed in the context of a previous study, DNA had been extracted and subjected to duplicate WGA as described (2). Genomic DNA from purified HCL cells was extracted using QIAmp DNA Mini Kit.

To confirm the clonal relationship between onset and relapsed specimens, IGHV-D-J gene rearrangements and GeneScan analysis were performed according to the BIOMED-2 protocols. 50 ng of gDNA from microdissected cells were subjected to BIOMED-2 multiplex PCRs for the immunoglobulin heavy chain FR2VHMIX-JH (tubes B) rearrangements. Positive (clonal) and negative (polyclonal) controls in addition to a blank control (H2O) were included in all runs. Fluorescence-labeled PCR products were separated by capillary electrophoresis, examined by a high-resolution fragment length analyzer (ABIPRISM 310; Applied Biosystems), and evaluated by GeneMapper 4.0 software (Applied Biosystems). Monoclonal gene rearrangements were identified as prominent, single-sized amplification products (3).

**Quantitative PCR Assay to measure EBV Genome Load**

Amplification reactions were performed in technical triplicates in a 50-μl volume reaction containing 25 μl TaqMan Universal master mix (Life Technologies Italia, Italy), forward and reverse primers (15 pmol each) and 10 pmol FAM-labeled TaqMan probes, specific for EBNA1, BamHI-W, HBB or ApoB (Cat.# 4482777, 4453320, 4448892, Life Technologies Italia, Italy), 100 ng of test DNA in 10 μl and water to the final volume. The reaction mixtures were amplified for 40 cycles (15’’ at 95°C, 60’’ at 60°C), after an initial activation (50°C for 2’ and a subsequent hold at 95°C for 10’). EBV viral load was calculated based on the ratio of the copies of the BamHI-W or EBNA1 to HBB in a given volume of extracted DNA, with HBB used as a denominator to obtain the copy number per cell equivalent. The resulting ratio was then multiplied by 10,000 to provide the number of copies of EBV per 10,000 cell equivalents. Each experiment included DNA samples from EBER-positive and EBER-negative cases, as well as water-only controls. Samples were considered negative if Ct values exceeded 40 cycles. The quantification results for experimental samples were extrapolated from the EBNA1 and BamHI-W calibration curves, after averaging the replicates (and also after further averaging of the results consistently obtained in each WGA duplicate of the 19 cHL cases from Perugia).

**Droplet digital PCR Assay to measure the absolute copy number of EBV Genome Load**

Droplets digital PCR (ddPCR) was performed using 150 ng of DNA, 1 × ddPCR Supermix for Probes (BioRad, Hercules, CA, USA), and the same primer used in qPCR with a concentration of 0.30 µM of each primer, and 0.6 µM of the probe in a total volume of 22  µL. The protocol applied for the detection of viral miRNAs was as follow: 150 ng of, 1 × ddPCR Supermix for Probes (BioRad, Hercules, CA, USA), of 2 µl of each primer (TaqMan, LifeTech), and 1 µl of each probe in a total volume of 22  µL. We prepared the samples in a 96 wells ddPCR plates and then 20 µl of each sample were loaded onto middle wells of a cartridges for droplet generation using QX200TM Droplet Generator instrument (BioRad), following manufacture ‘instructions. Only 8 samples need to be loaded onto a cartridge at any one time, filling with 70 µl of generator oil to the bottom wells of the cartridge, while top wells were empty to receive generated droplets. When droplet generation was complete, top wells of cartridge contained droplets, with middle and lower wells nearly empty. 40 µl the generated microdroplets were put into a 96-Well Semi-Skirted PCR plate for amplification. We repeated these steps as needed to complete our samples. Cycling conditions included preheating at 95 °C for 10 min followed by 40 cycles of denaturation at 94 °C for 30 s, annealing at 58 °C for 60 s, and final heating at 98 °C for 10 min. Then, the PCR plate was transferred to a QX100 droplet reader (BioRad), and fluorescence amplitude data were obtained by QuantaSoft software (BioRad). The absolute copy number of each viral assays was calculated by Bio-Rad software and showed as number of copies/µl. Some minor modifications were made to calculate the number of copies per 10,000 cells. In particular, we calculated the number of viral copies per single cell as viral copies/(HBB copies/2)(4,5) and then, we arbitrarily multiplied per 10,000, in order to make a comparison between ddPCR and qPCR results.

**Quantitative Reverse Transcription-PCR Assay to measure Viral miRNAs**

FFPE lymphoma tissue sections stained for EBER-ISH were used for a separate round of tumour cell microdissection performed exactly as described for DNA qPCR, with the following modifications to preserve RNA. RNAse contamination was avoided by cleaning surfaces of the microscope, slide, bench and other tools with RNAse Zap solution (Ambion, Carlsbad, Ca). The slide and reagent preparation for LCM was performed as described in Butler *et al,* 2016 (6). Briefly, RNAse-free glass coplin jars were prepared for staining by rinsing with 100% ethanol, followed by distilled water, RNAse Zap solution followed again by RNAse-free water. RNase inhibitor (ProtectRNA™ 500×, R7397; Sigma, St. Louis, MO) was added to the staining solutions. Microdissected cells were catapulted in a tube filled with 10 ul of extraction buffer (PicoPure RNA isolation Kit) and 0.5 ul of RNAse inhibitor (1U/ul) (Ambion, AM2694, Carlsba, Ca) and the RNA was extracted following the manufacturer’s instructions and quantified by Nanodrop spectrophotometer (NanoDrop Technologies LLC, USA). Total RNA (ranging from 160-230) ng was extracted from approximately 50’000 non-Hodgkin lymphoma cells and 10 ng of RNA per reaction were reverse transcribed by using TaqMan primers against three EBV-encoded miRNAs, namely EBV-miR-BART9-5p, EBV-miR-BART10-3p, and EBV-miR-BART19-3p (TaqMan assays Cat. # 006884, 004421_mat, 197235_mat,) in three separate reactions as described (5). Then, TaqMan probes specific for each selected viral miRNA were added to analyse by qPCR all cases in technical triplicates. RNU6B was used as endogenous control (Applied Biosystems, Applera, Italy) and the viral niRNAs expression was calculated using the 2^-Δct^ formula applied to the replicates’ mean. Kruskal–Wallis Test was applied for statistical analysis. miRNAs quantification was not assessed in HLs cases because of the poor quantity of RNA obtained from the Reed-Sternberg cells.

**Droplet digital PCR Assay to measure the absolute copy number of viral miRNAs**

Droplets digital PCR (ddPCR) was performed to detect the copies of viral miRNAs present in those cases initially EBER-negative but resulted qPCR-posiitve. The protocol applied was as follow: 150 ng of RNA, 1 × ddPCR Supermix for Probes (BioRad, Hercules, CA, USA), of 2 µl of each primer (TaqMan, LifeTech), and 1 µl of each probe in a total volume of 22  µL. 20ul of each samples were loaded onto middle wells of a cartridges for droplet generation and then transfer into a 96-Well Semi-Skirted PCR plate for amplification. for droplet generation using QX200TM Droplet Generator instrument (BioRad), following manufacture ‘instructions. Only 8 samples need to be loaded onto a cartridge at any one time, filling with 70 µl of generator oil to the bottom wells of the cartridge, while top wells were empty to receive generated droplets. When droplet generation was complete, top wells of cartridge contained droplets, with middle and lower wells nearly empty. 40 µl the generated microdroplets were put into a 96-Well Semi-Skirted PCR plate for amplification. We repeated these steps as needed to complete our samples. Cycling conditions included preheating at 95 °C for 10 min followed by 40 cycles of denaturation at 94 °C for 30 s, annealing at 58 °C for 60 s, and final heating at 98 °C for 10 min. Then, the PCR plate was transferred to a QX100 droplet reader (BioRad), and fluorescence amplitude data were obtained by QuantaSoft software (BioRad). The absolute copy number of each viral assays was calculated by Bio-Rad software and showed as number of copies/µl, that subsequently was multiplied for the final volume of the PCR reaction (22 µl), normalized to the housekeeping loading control (RNU6B) and expressed in each case as number of viral miRNA copies/10,000 RNU6B copies as well as percentage of the corresponding average value across conventional EBV-positive cases of the same histology.

**RNAscope for EBNA1 mRNA**

RNA *in situ* hybridization was performed using the RNAscope 2.0 HD Red Chromogenic Reagent Kit (Advanced Cell Diagnostics, CA) and V-EBV-EBNA1 (Advanced Cell Diagnostic, CA) target probe, according to the manufacturer’s instructions. Each sample was quality-controlled for RNA integrity with a probe specific to the housekeeping *PPIB* mRNA used as positive control. Briefly, sections of FFPE tissue were baked for 1 hr at 60°C prior to use. After deparaffinization and dehydration, the tissues were air-dried and treated with a peroxidase blocker before boiling in Target Retrieval Reagent solution for 15 min. Protease Plus was then applied for 30 min at 40°C. Target probes (for viral EBNA1 mRNA, cellular PP1B mRNA as housekeeping positive control, and bacterial *DapB* gene as negative control were hybridized for 2 hrs at 40°C, followed by a series of signal amplification steps (AMP1-6), each separated by two washes in the washing buffer (provided by the kit). Signal detection was performed by hybridizing the FastRed probe mix (RED- A and RED-B) on each sample and counterstaining the section with haematoxylin. RNA staining signals were identified as red punctate dots. Background staining was evaluated using a negative control probe specific for bacterial dihydrodipicolinate reductase (*dapB)*; all lymphoma cases analysed did not show any dots for *dapB* in any cells.

**Methylation studies**

Genomic DNA was extracted from five 5-µm-thick whole sections of FFPE non-Hodgkin lymphoma sections using the NucleoSpin Tissue extraction kit (Macherey-Nagel, Germany) according to the manufacturer’s instructions. The amount and quality of DNA were evaluated by measuring the optical density (OD) at 260 nm, the 260/230 and the 260/280 ratios using a Nanodrop spectrophotometer (NanoDrop Technologies LLT, USA).

300 ng of DNA from each sample were used for bisulfite conversion, in which unmethylated cytosine was converted to uracil with the EpiTect Fast DNA bisulfite kit (Qiagen, Hilden, Germany, 59824) according to the manufacturer's instructions. Human HCT116 DKO Non-methylated and Methylated DNA (Zymo Research, USA, D5014-1/2) were used as standards controls. Briefly, DNA was mixed with RNAse-free water. Bisulfite solution and DNA protector buffer and the conversion were run on an PTC-200 Peltier Thermocycler (MJResearch, USA) at the following conditions: Denaturation for 5 min at 95°C, Incubation for 10 min at 60°C, Denaturation for 5 min at 95°C, Incubation for 10 min at 60°C. Converted DNA was purified following the manufacture’s instructiors and then eluted in 15 µL of elution buffer (EB). Then, PCR was performed using bisulfite-converted DNA and the PyroMark PCR MGMT kit (Qiagen, Hilden, Germany). Primer sets with one biotin-labeled primer were used to amplify the bisulfite-converted DNA samples. *CDH1* primers were purchased from Qiagen (Milan, Italy) while *MGMT* primers from Diatech. Briefly, regarding *CDH1* gene, the mix was composed by 12.5 µL master mix, 1,25 µL Eva Green, 0,2 µM of primer, 2,5 mM MgCl2, 5,75 µL of water, and 2 µL sample were mixed for each reaction and run under the following thermal cycling conditions: initial activation at 95°C for 15 minutes, followed by 45 cycles of 30 seconds at 94°C, 30 seconds at the optimized primer-specific annealing at 56°C, and 30 seconds at 72°C, followed by a extension for 10 minutes at 72°C. The PyroMark Q96 CpG *MGMT* plus kit (Diatech, Italy) is available as a ready-to-use research kit. The PCR conditions were the following: initial activation at 95°C for 5 minutes, followed by 45 cycles of 30 seconds at 95°C, 30 seconds at the optimized primer-specific annealing at 53°C, and 20 seconds at 72°C, followed by an extension for 5 minutes at 72°C. In both condition, amplification of the correct DNA product was confirmed by acquiring the green fluorescence signal with 5 final cycles of 20 seconds at 60°C. The reaction was performed by using Rotor-Gene 6000. Streptavidin beads (3 µL; GE Healthcare, Buckinghamshire, UK), 40 µL PyroMark binding buffer (Qiagen), 20 µL PCR product, and 17 µL water were mixed and incubated for at least 10 minutes on a shaking table at 1400 rpm. Amplicons were separated, denatured, washed, and added to a plate containing annealing buffer and primers (36 µL and 4 µL for CDH1; 38 µL and 2 µL for MGMT) per well using the PyroMark Q96 Vacuum Workstation (Qiagen). Primer annealing was performed by incubating the samples at 80°C for 2 minutes and then cooling to room temperature prior to pyrosequencing. The signal and target CpGs were evaluated by was analyzed using the PyroMark Q96 ID System which converts the pyrograms to numerical values for peak heights and calculates the proportion of methylation at each base as a C/T ratio. Along with standards, a cytosine not followed by a guanine, which was not methylated, served as an internal control to verify the efficiency of bisulfite conversion.

**REFERENCES**

1. Abate F, *et al*. Disctinct viral and mutational spectrum of endemic Burkitt lymphoma. *PLoS Pathog*. 2015;11(10): e1005158.
2. Tiacci E, Ladewig E, Schiavoni G, et al. Pervasive mutations of JAK-STAT pathway genes in classical Hodgkin lymphoma. *Blood*. 2018;131(22): 2454-2465.
3. van Dongen JJ, *et al*. Design and standardization of PCR primers and protocols for detection of clonal immunoglobulin and T-cell receptor gene recombinations in suspect lymphoproliferations: report of the BIOMED-2 Concerted Action BMH4-CT98-3936. *Leukemia*. 2003;17(12):2257-2317.
4. Lillsunde Larsson G, Helenius G. Digital droplet PCR (ddPCR) for the detection and quantification of HPV 16, 18, 33 and 45 - a short report. Cell Oncol (Dordr). 2017; 40:521-527.
5. Mundo L, *et al*. Unveiling another missing piece in EBV-driven lymphomagenesis: EBV- encoded microRNAs expression in EBER-negative Burkitt lymphoma cases. *Front Microbiol*. 2017;8: 229.
6. Butler AE, Matveyenko AV, Kirakossian D, Park J, Gurlo T, Butler PC. Recovery of high- quality RNA from laser capture microdissected human and rodent pancreas. *J Histotechnol*. 2016;39(2): 59-65.

SUPPLEMENTARY FIGURE LEGENDS

**Supplementary Figure 1. Detection of EBV genome by Droplets Digital PCR in lymphoma cell lines.** Conventional EBV-positive cell lines of cHL (panel A; L591 and AM-HLH) and BL (panel A; Namalwa) showed a high copy number for both viral assays (average of 6090 EBNA1 copies and 2430 BamHI-W copies per 10,000 cells, in right and left part of each panel respectively). Conversely, low ddPCR signals for one or both viral genes were detected in 6/7 EBV-negative HL cell lines (panel A; KMH2, SUP-HD1, UHO1, L540, HDLM2, L1236) and in 4/5 EBV-negative BL cell lines (panel B; AKATA-2A8, BL41, Ramos, BL2). No ddPCR signals were observed in two conventional EBV-negative cell lines of cHL (L428, panel A) and BL (DG75, panel B) and in control cell lines of acute myeloid leukemia (Kasumi, TF1, KG1, OCI-AML3, U937) and multiple myeloma (CAG, RPM1).

**Supplementary Figure 2. Different EBNA1 staining pattern in reactive EBV-infected lymphocytes compared to tumor cells of conventional EBV-negative lymphomas showing traces of EBV infection.** *(Top panel)* In this EBNA1-RNAscope staining of a lymph node section from a conventional EBV-negative cHL case scoring positive at qPCR and ddPCR for EBNA1, a tumor cell shows faint punctuate labeling whrereas an occasional bystander small lymphocyte displays strong homogeneous labeling. *(Bottom panel)*. Similarly, EBNA1-RNAscope staining of an EBV-infected reactive germinal center shows a mostly strong staining in centroblasts and centrocytes.
